# Phenotypic and Genomic Properties of *Chitinispirillum alkaliphilum* gen. nov., sp. nov., A Haloalkaliphilic Anaerobic Chitinolytic Bacterium Representing a Novel Class in the Phylum *Fibrobacteres*

**DOI:** 10.3389/fmicb.2016.00407

**Published:** 2016-03-31

**Authors:** Dimitry Y. Sorokin, Andrey L. Rakitin, Vadim M. Gumerov, Alexey V. Beletsky, Jaap S. Sinninghe Damsté, Andrey V. Mardanov, Nikolai V. Ravin

**Affiliations:** ^1^Winogradsky Institute of Microbiology, Research Center of Biotechnology of the Russian Academy of SciencesMoscow, Russia; ^2^Department of Biotechnology, Delft University of TechnologyDelft, Netherlands; ^3^Institute of Bioengineering, Research Center of Biotechnology of the Russian Academy of SciencesMoscow, Russia; ^4^Department of Marine Organic Biogeochemistry, NIOZ Royal Netherlands Institute for Sea Research and and Utrecht UniversityUtrecht, Netherlands; ^5^Geochemistry, Department of Earth Sciences, Faculty of Geosciences, Utrecht UniversityUtrecht, Netherlands

**Keywords:** Fibrobacteres, candidate phylum TG3, haloalkaliphilic bacterium, chitin, genome sequence

## Abstract

Anaerobic enrichment from sediments of hypersaline alkaline lakes in Wadi el Natrun (Egypt) with chitin resulted in the isolation of a fermentative haloalkaliphilic bacterium, strain ACht6-1, growing exclusively with insoluble chitin as the substrate in a sodium carbonate-based medium at pH 8.5–10.5 and total Na^+^ concentrations from 0.4 to 1.75 M. The isolate had a Gram-negative cell wall and formed lipid cysts in old cultures. The chitinolytic activity was associated with cells. Analysis of the 4.4 Mb draft genome identified pathways for chitin utilization, particularly, secreted chitinases linked to the cell surface, as well as genes for the hydrolysis of other polysaccharides and fermentation of sugars, while the genes needed for aerobic and anaerobic respiration were absent. Adaptation to a haloalkaliphilic lifestyle was reflected by the gene repertoire encoding sodium rather than proton-dependent membrane-bound ion pumps, including the Rnf-type complex, oxaloacetate decarboxylase, V-type ATPase, and pyrophosphatase. The phylogenetic analysis using 16S rRNA gene and ribosomal proteins indicated that ACht6-1 forms a novel deep lineage at the class level within the bacterial candidate division TG3. Based on phylogenetic, phenotypic and genomic analyses, the novel chitinolytic bacterium is described as *Chitinispirillum alkaliphilum* gen. nov., sp. nov., within a novel class *Chitinispirillia* that could be included into the phylum *Fibrobacteres.*

## Introduction

Chitin forms the skeleton of most insects and crustaceans, and is an important structural component of the fungal cell wall. Chitin is an analog of cellulose in its structural and mechanistic properties. Complete chitin depolymerization demands the action of exo- and endo-chitinases and chitobiohydrolase (Gooday, 1990; Karlsson and Stenlid, 2008), similar to enzymatic cellulose degradation. The genes encoding various chitinases are present in many sequenced microbial genomes, although the number of prokaryotic species utilizing insoluble chitin as a growth substrate and with characterized chitinolytic systems is still limited (Stoykov et al., 2015; Bai et al., 2016). Even less is known about microbial chitin utilization under high salt and high pH conditions, where the massive development of brine shrimp *Artemia* makes chitin a possible most abundant biopolymer in hypersaline brines (Liaw and Mah, 1992; Hau-Heredia et al., 2009). The massive presence of *Artemia* has been documented in haloalkaline Mono Lake in California (Dana and Lenz, 1986), haloalkaline lakes in Wadi el Natrun in Egypt (El-Bermawi et al., 2004) and also noted in the hypersaline soda lakes of south-western Siberia (Sorokin et al., 2012). So far, the potential to grow with chitin at salinity up to 2 M NaCl has been described for two fermentative bacteria from the order *Halanaerobiales*, *Halanaerobacter chitinivorans* (Liaw and Mah, 1992), and *Orenia chitinitropha* (Sorokin and Kolganova, 2014), and only recently a report on the ability of haloarchaea to use chitin as growth substrate has appeared (Hou et al., 2014; Sorokin et al., 2015).

Evidence of chitin utilization under extremely haloalkaline conditions was also obtained only recently during our investigation of hypersaline soda lakes which resulted in the isolation of a number of chitinotrophic haloalkaliphilic bacteria capable of optimal growth with insoluble chitin at pH 10 in soda brines (Sorokin et al., 2012). The most active chitin utilization was found under anaerobic conditions by two different groups of fermentative haloalkaliphilic bacteria which were unique in their exclusive specialization on insoluble chitin as their growth substrate. Both groups belong to the candidate phylum-level division TG3 (Termite Group 3), and they represented the first culturable members of its two subphyla. The candidate phylum TG3 includes numerous clones from the guts of higher termites, deep-sea sediments, and soils (Hongoh et al., 2005, 2006). Association with the anoxic termite gut segments suggested that these bacteria are involved in anaerobic polymer degradation, particularly cellulosic substrates (Hongoh et al., 2006; Hongoh, 2010), but this was not demonstrated until now since no isolates were obtained. An extremely salt-tolerant group of haloalkaliphilic anaerobic chitinolytics has recently been described as a novel genus and species *Chitinivibrio alkaliphilus* (*Chv. alkaliphilus*) within a novel class *Chitinivibrionia*, and the genome analysis results have also been published recently (Sorokin et al., 2014).

In this work, we describe the phenotypic and genomic characteristics of a representative of the second, low-salt tolerant group of alkaliphilic anaerobic chitin-utilizing bacteria isolated from hypersaline alkaline lakes in Egypt. The bacterium forms a second deep lineage of the class level in the candidate phylum TG3 and is proposed as a novel genus and species within a novel class.

## Materials and Methods

### Samples Collection, Enrichment, and Cultivation Conditions

A composite sediment sample obtained from seven hypersaline alkaline lakes in Wadi el Natrun valley (Libyan desert, Egypt; N30°24′/E30°18′) in September 2001 was used as an inoculum. There is a chain of 6–7 shallow hypersaline lakes with salinity within a range from 200 to 360 g/L dominated by NaCl with a fraction of sodium carbonates ranging from 0.1 to 1 M total carbonate alkalinity, resulting in brine pH of 9 to 10 (Imhoff et al., 1979; Taher, 1999). The cores were taken from the top 10 cm sediments by a plastic corer with 30 mm diameter and placed into glass bottles up to the top. The bottles were closed with a rubber stoppers and screw caps without air bubbles and placed into insulated sample container with chilling elements. After transportation to Moscow, the samples were kept at 4°C.

Strain ACht6-1 was enriched and cultivated at 30°C on a mineral medium based on Na carbonate buffer (pH 10) with 0.6 M Na^+^ in total and 0.5 g l^-1^ of K_2_HPO_4_. 1 mM MgSO_4_, 10 mg l^-1^ yeast extract and 1 ml l^-1^ each of acidic trace metal solution and vitamin mix (Pfennig and Lippert, 1966) were added to the sterilized medium. Crab shell chitin powder (Sigma) was soaked and washed several times in 0.1 M NaOH, then washed in water and sterilized. Amorphous chitin was prepared as described previously (Sorokin et al., 2012). Chitin was added to the growth medium to 1 g l^-1^. The influence of pH on growth rate was analyzed at 0.6 M Na^+^. The following buffer systems were used: 0.1 M HEPES and NaCl/NaHCO_3_ (pH 6–8), and sodium bicarbonate/sodium carbonate with 0.1 M NaCl (pH 8.5–11.0). The pH values at the end of exponential growth phase were recorded. To study the impact of concentration of soda on the growth rate, we used sodium carbonate medium (pH 10) with 0.2–3.0 M of total Na^+^ and amorphous chitin as substrate. Experiments on growth rate measurements were made in duplicate. The purity of the isolate was confirmed by 16S rRNA gene sequencing.

Utilization of different substrates by strain ACht6-1 was tested at standard growth conditions (0.6 M Na^+^, pH 10, 30°C) with 1 g/L of the following compounds: glucosamine, *N*-acetyl-glucosamine, C4–C6 chitin olygomers (a gift from Dr. S. Lopatin), D-glucose, D-fructose, D-xylose, D-ribose, D-galactose, sucrose, D-lactose, D-mannose, trehalose, D-cellobiose, D-galacturonic acid, birch wood xylane, lichenan, amorphous cellulose, filter paper, apple pectin, alginate, casein, bovine serum albumine, peptone from casein, soyton, yeast extract. For inoculum, the culture was grown on chitin until complete polymer utilization, the cells were harvested by centrifugation under anoxic conditions, washed once with the anoxic mineral medium and resuspended at the cell density ×50 times of the original culture. The concentrated cells were injected into prepared substrate range test flasks by syringe.

### Analytical Procedures

Analyses of isolate growth was performed by measuring an increase in optical density at 600 nm after extensive homogenization of the cultures followed by chitin removal first by gravity and then by centrifugation at 1000 × rpm for 10 s. After the OD measurements, cells were precipitated by centrifugation at 5000 g for total protein measurement (Lowry et al., 1951). The products of fermentation were measured by high performance liquid chromatography using BioRad HPX-87-H column (eluent 5 mM H_2_SO_4_ at 0.6 ml min^-1^; UV and RI detectors; 60°C). The production of H_2_ was analyzed by gas chromatography (GC). Phase contrast microphotographs were acquired using a Zeiss Axioplan Imaging 2 microscope (Göttingen, Germany). For total-cell electron microscopy analysis, the bacteria were collected and transferred to neutral NaCl buffers with the same salinity as in the growth medium, fixed using glutaraldehyde and positively stained with uranyl acetate. For thin section analysis, the cells were fixed with OsO_4_, stained in uranyl acetate, washed in ethanol, embedded in Epon resin and stained with lead citrate after sectioning.

The cells for the membrane lipid analysis were grown at standard conditions (0.6 M Na^+^, pH 10, 30°C) until full consumption of chitin. The cells were harvested by centrifugation, washed once in 0.5 M NaCl and freeze dried. 1 N HCl in methanol was used to hydrolyze the lyophilized cells. The pH of hydrolysate was adjusted to 4.0 with 2 N KOH-methanol (1:1, vol/vol). Upon adding of water to a final 1:1 ratio of H_2_O–MeOH, the hydrolysate was three times extracted with dichloromethane (DCM). The extract was dried over Na_2_SO_4_, methylated with diazomethane and separated into an apolar and a polar fraction on activated Al_2_O_3_ column using DCM and DCM-MeOH (1:1, vol/vol) as the eluent. The polar lipids fraction was dissolved in hexane–2-propanol (99:1, vol/vol) and analyzed for branched GDGTs by HPLC–atmospheric pressure chemical ionization mass spectrometry (Sinninghe Damsté et al., 2011). The apolar lipids fraction containing the fatty acid methyl esters was characterized by GC and GC-mass spectrometry. Double-bound positions of the monounsaturated fatty acid methyl esters were identified on the basis of the mass spectra of their dimethyl disulfide derivatives.

### Genome Sequencing and Annotation

Whole-genome sequencing was carried out employing the GS FLX genome sequencer (Roche). A shotgun genome library was sequenced following the Titanium protocol on one half of a picotiter plate, resulting in 334162 reads with an average length of 554 bp. The reads obtained in pyrosequencing reactions were *de novo* assembled into contigs using GS *De Novo* Assembler v. 2.6 software.

Contigs longer than 500 bp were used for gene prediction and annotation. Transfer RNA (tRNA) genes were identified using tRNAscan-SE (Lowe and Eddy, 1997). Whole-genome annotation was carried out with the RAST service (Aziz et al., 2008), with subsequent manual inspection. SignalP v. 3.0 was used to identify the signal peptides (Bendtsen et al., 2004). The annotated sequence of the ACht6-1 genome has been submitted to the GenBank database under accession number LDWW00000000.

### Phylogenetic Analysis

The 16S RNA sequences were aligned using MUSCLE included in MEGA 5.05 (Tamura et al., 2011). Before the phylogenetic reconstruction, ambiguously aligned sites were removed using Gblocks (Talavera and Castresana, 2007). The maximum likelihood phylogenetic tree was computed by MEGA 5.05, using General Time Reversible substitution model and uniform rates among sites. Bootstrap tests were performed with 100 resamplings.

For phylogenetic analysis of ribosomal proteins the following 31 proteins were used: L1, L2, L3, L4, L5, L6, L11, L13, L14, L16, L17, L18, L19, L20, L22, L27, S2, S3, S4, S5, S7, S8, S9, S10, S11, S12, S13, S15, S17, S18, and S19. Alignment of concatenated sequences of the ribosomal proteins was performed using MUSCLE software included in MEGA 5.05 package. Before the phylogenetic reconstruction poorly aligned sites have been removed using Gblocks. The maximum likelihood phylogenetic trees were constructed by RAxML v7.3.5 (Stamatakis, 2006), using the gamma model of rate heterogeneity and JTT substitution matrix. The bootstrap support values were estimated from 100 replicates.

All predicted ACht6-1 proteins were screened against NCBI NR database using BLASTP. The proteins that revealed a significant hit (*e*-value < 1*e*-10) were distributed between two groups: proteins that could be assigned a tentative function, and proteins that matched only to those with unknown function. Proteins without significant BLASTP hits in the NCBI database were considered as strain-specific.

### Hydrolytic Activity Assays

Qualitative analysis was performed by agar-diffusion method. Cells grown on chitin were transferred to soda buffer with pH 10 and 0.6 M Na^+^ and stored frozen. Before the test the cells were disrupted by repeated freeze-sawing. Carboxymethyl cellulose (Fluka, 21900), birch-wood xylan (Sigma, X0502), beech-wood xylan (Serva, 38500), soluble starch (Sigma, S2004), and skim milk powder (Sigma, 70166) were used as substrates in 1% agarose plates prepared with mineral soda buffer (pH 10, 0.4 M total Na^+^).

Quantitative chitinolytic activities of whole cells of ACht6-1 strain, culture supernatants and extracts of lysed cells were determined in a fluorimetric assay with derivatives of 4-methylumbelliferyl (4-MU) employing a Chitinase Assay Kit (Sigma, CS1030). 4-MU-diacetyl-β-D-chitobioside and 4-MU-*N*-acetyl-β-D-glucosaminide were used for detection of chitobiosidase and β-*N*-acetylglucosaminidase activities, respectively, while 4-MU-β-D-*N*,*N*′,*N*″-triacetylchitotriose was employed for measurement of endochitinase activity. Hydrolytic activities were assayed in 25 mM Na-phosphate buffer (pH 8.0) at 30°C.

The hydrolytic activities of ACht6-1 lysed cell extracts with amorphous chitin, carboxymethyl cellulose (Fluka, 21900), microcrystalline cellulose (Aldrich, 310697), starch (Sigma, S2004), birch-wood xylan (Sigma, X0502), beech-wood xylan (Serva, 38500), and chitosan (200 kDa, ZAO Bioprogress, Russia) were measured using the DNS method (Miller, 1959). Assays were performed in 25 mM Na-phosphate buffer (pH 8.0) with 0.5 M NaCl at 30°C. Substrates were used at the following concentrations: chitin, 1% w/v; chitosan, 2% w/v; starch, 2% w/v; carboxymethyl cellulose, 2% w/v; microcrystalline cellulose, 2% w/v; birch-wood xylan, 1.5% w/v; beech-wood xylan, 1.5% w/v.

## Results and Discussion

### Isolation and Phenotypic Characterization of Strain ACht6-1

**FIGURE 1:**
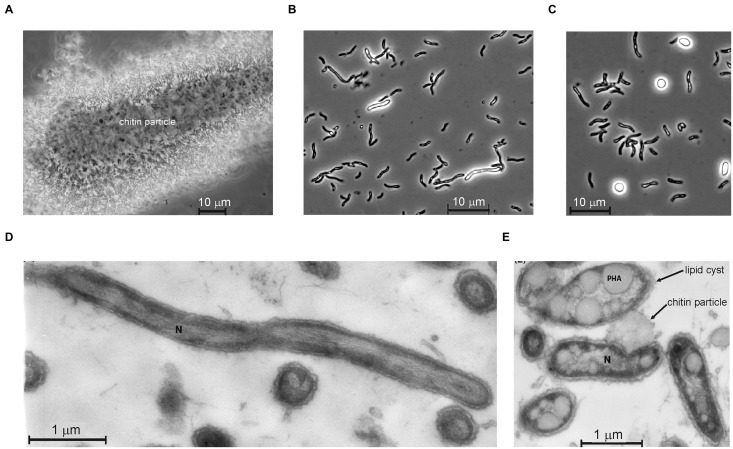
**Cell morphology of strain ACht6-1 grown on amorphous chitin at pH 10 and 0.6 M Na^+^.** Phase contrast microphotographs showing ACht6-1 cells bound to a chitin particle **(A)** and initial **(B)** and final **(C)** stages of the lipid cyst formation in free cells. Thin section electron microscopy showing ultrastructural organization of cells of strain ACht6-1 growing on chitin: actively growing cells from the exponential phase **(D)** and cells from the stationary phase full of PHA lipid storage **(E)**. The image in panel C was modified from Figure 5 in Sorokin et al. (2012).

A composite surface sediment sample from seven hypersaline alkaline lakes in Wadi el Natrun (Libyan Desert, Egypt) was used for the isolation of anaerobic chitinolytic bacteria (Sorokin et al., 2012). The pH in the soda brines was between 9.4 and 10.1, the salinity varied between 200 and 360 g l^-1^ and the carbonate alkalinity varied from 0.1 to 0.75 M (Sorokin et al., 2012). Anaerobic enrichments with crystalline chitin inoculated with mixed anoxic sediments at 0.6 M of total sodium and pH 10 were dominated by two vibrio-shaped bacteria differing in size. Subculturing at 2 M total Na^+^ separated the small sized cells (strain ACht6-2), described previously as a member of the genus *Chitinivibrio* (Sorokin et al., 2014), while the larger spirillum-shaped cells grew faster at moderate salinity and were eventually isolated in pure culture by the dilution to extinction technique at 0.6 M Na^+^ and designated as strain ACht6-1. The isolate contained spirilloid cells motile with a single polar flagellum. In the initial growth phase, the cells were mostly attached to chitin particles. After visible chitin degradation, the cells started to massively detach from the solid phase into the liquid phase and to form round refractive bodies consisting mostly of PHB-like (Nile Blue staining) granules (**Figure 1**). Whilst complete chitin depolymeriszation occurred, massive cell lysis commenced, similar to that previously described for the haloalkaliphilic chitinotrophic anaerobe *Chtinivibrio* (Sorokin et al., 2012, 2014). It is well-documented that in Gram-negative bacteria, massive autolysis can be triggered under certain conditions, mostly in the stationary phase when the cells are losing control of their autolysins, which also include a lysozyme-like member (Holtje, 1995; Shockman et al., 1996). In our case, it might be speculated that when the substrate (chitin) is consumed, the cell-bound chitinases start to digest the cell wall murein layer of ACht6-1.

Polar lipid analysis of strain ACht6-1 showed the presence of seven species of which two cannot be identified, while the identifiable species were the following, in order of abundance: ornithine lipid, phosphoglycerol (three different modifications) and phosphohexose. The PLFA profile of ACht6-1 was dominated by aiC15:0 and C16:0, with C14:0 and iC16:0 as sub-dominants (see **Table S1** in the Supplemental Material).

**FIGURE 2:**
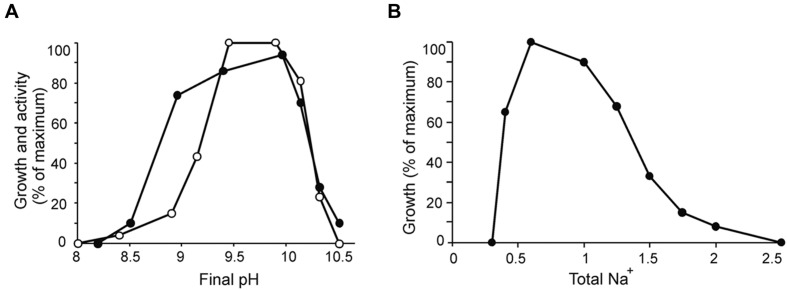
**Influence of pH at 0.6 M total Na^+^**(A)** and salinity at pH 10 **(B)** on growth (closed circles) and chitinolytic activity (open circles) of ACht6-1 cells.** For the activity test, a cell suspension (protein concentration 0.15 mg/ml) was incubated anaerobically in sodium bicarbonate–carbonate buffer with 3 mg/ml insoluble amorphous chitin on a rotary shaker at 100 rpm and at 30°C for 72 h. At the end of experiment, the cells and residual chitin were separated from the supernatant by centrifugation, the pellet was resuspended in 1 ml of 1 M NaOH and incubated at 45°C for 1 h to digest the cells. The chitin degradation was measured by optical density at 600 nm in comparison with the starting conditions. The results are mean from duplicate experiments.

Strain ACht6-1 was an obligate alkaliphile growing at pH from 8.5 to 10.5 (optimum at 9.5–10). It is relatively tolerant to salinity growing optimally in sodium carbonate buffer containing 0.5–1 M total Na^+^ (**Figure 2**). Chloride ions were not required for growth as is typical for most of natronophilic bacteria isolated from soda lakes.

Similar to the representatives of the genus *Chitinivibrio*, strain ACht6-1 utilized chitin as the only growth substrate. Neither monomer *N*-acetyl glucosamine (GlcNAc), nor soluble chitin oligomers, supported growth. No growth was observed on chitosan, various forms of cellulose, starch, xylans, several types of glucans, pectin or proteins and other tested substrates indicated in M&M section. The type of metabolism was obligatory fermentative, with products including H_2_ in the gas phase and acetate, glycerol, ethanol, and formate (in order of abundance on the basis of Cmol) in the liquid phase. The maximal growth rate of ACht6-1 at optimal pH and salinity was 0.19 h^-1^ and the growth yield in the exponential phase was 0.35(±0.05) moles of biomass carbon per mole of chitin carbon. Hydrolysis of insoluble chitin was not observed in culture supernatants, but was detected during incubations of the intact cells washed from the medium and transferred to soda buffers, indicating that the activity is cell-bound. However, contrary to *Chv. alkaliphilus* ACht-1, the chitinolytic activity of ACht6-1 was not abolished by cell lysis.

The exo- and endo-chitinase activities of ACht6-1 with synthetic soluble substrates were also cell-linked and were not reduced upon cell lysis by sonication. Maximal activity was detected with β-*N*-acetylglucosaminidase substrate 4-MU-*N*-acetyl-β-D-glucosaminide, while about 100-fold lower activities were observed with 4-MU-diacetyl-β-D-chitobioside and 4-MU-β-D-*N*, *N*′,*N*″-triacetylchitotriose. This pattern is quite different from that observed in *Chv. alkaliphilus*, for which the maximal activity was detected with 4-MU-β-D-*N*, *N*′,*N*″-triacetylchitotriose (Sorokin et al., 2014). Maximum endochitinase activity was observed at about 0.5 M Na^+^ indicating its extracellular localization (see **Figure S1** in the Supplemental Material). On the contrary, β-*N*-acetylglucosaminidase activity was maximal at low salinity and slightly increased upon cell lysis (see **Figure S1** in the Supplemental Material), suggesting that the corresponding enzymes might act both inside and outside of the cell.

### General Features of the Genome

A draft genome of strain ACht6-1 was assembled into 160 contigs longer than 200 bp with N50 contig length of 123925 bp. The genome of ACht6-1 is about 4.43 Mbp long with the GC content of 42.9%. Three copies of 16S-23S-5S rRNA operon and 43 tRNA genes coding for all of the 20 amino acids were identified. Annotation of the genome sequence revealed 3,775 potential protein-coding genes of which 2,288 (61%) can be functionally assigned; 854 genes are unique to ACht6-1 with no significant similarity to any known sequences. Analysis of conserved single-copy marker genes using a set of 111 genes (McLean et al., 2013) revealed that the assembly includes 110 of these genes indicating completeness of the genome (a single missing gene TIGR00388 could be absent in some Bacteria). A search for potential genes in contigs shorter than 200 bp revealed only fragments of CDSs related to mobile elements or encoding hypothetical peptides with no significant hits in the NCBI database, indicating that the analysis of contigs longer than 200 bp provided comprehensive information on ACht6-1 genome.

In agreement with cell morphology, the ACht6-1 genome contains a set of genes involved in flagellation and chemotaxis regulation, as well as the genes for type IV pili-based twitching motility. These pilin proteins might be used by ACht6-1 to attach to chitin particles similar to some cellulolytic bacteria, such as *Melioribacter roseus* and *Fibrobacter succinogenes*, adhering to cellulose (Jun et al., 2007; Kadnikov et al., 2013).

Analysis of the ACht6-1 genome revealed that it could produce glycine betaine as an osmolyte for protection from high salinity. Genes encoding choline dehydrogenase (CHISP_0519) and betaine aldehyde dehydrogenase (CHISP_2272) were identified. Enzymes for three pathways in the synthesis of another compatible solute, trehalose, were encoded: trehalose-6-phosphate synthase OtsA and trehalose-6-phosphate phosphatase OtsB, malto-oligosyltrehalose synthase TreY and glycosyltrehalose trehalohydrolase TreZ, and trehalose synthase TreS. Genes related to ectoine biosynthesis, another compatible solute commonly found in halophilic and halotolerant bacteria, including *Chv. alkaliphilus*, were not identified.

### Phylogenetic Placement of Strain ACht6-1

**FIGURE 3:**
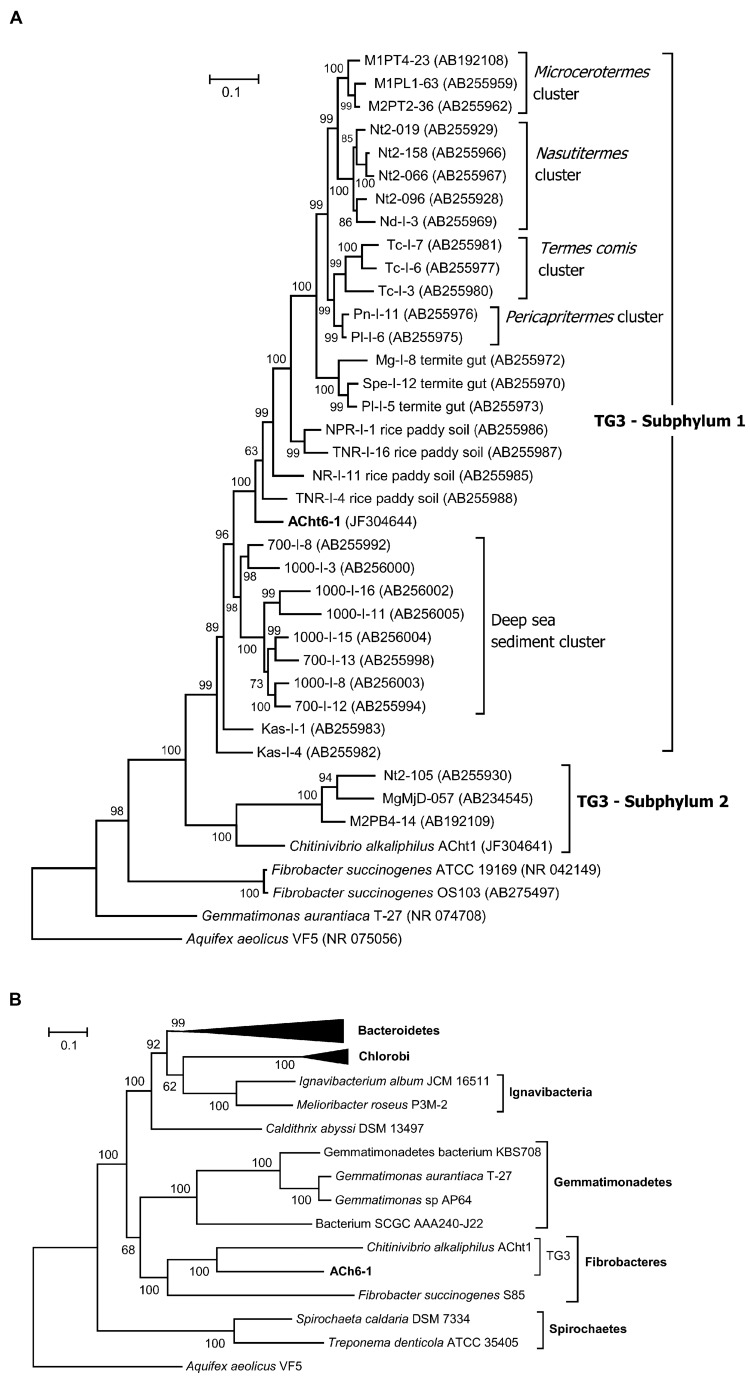
**Phylogenetic position of the ACht6-1 strain.** **(A)** Maximum likelihood tree based on the 16S rRNA gene sequences. **(B)** Maximum likelihood tree based on the concatenation of 31 ribosomal proteins. The 16S RNA or ribosomal protein sequences from *Aquifex aeolicus* VF5 were used as an outgroup. Numbers at nodes represent bootstrap values (100 replications of the original dataset), only numbers above 50% are shown. The scale bar represents the average number of substitutions per site.

A search of the ACht6-1 16S rRNA gene against the SILVA ribosomal RNA gene database (Quast et al., 2013) showed that the closest cultivated relative of ACht6-1 is *Chv. alkaliphilus* ACht1 (81.8% identity of 16S rRNA sequences), the representative of class *Chitinivibrionia* in the candidate phylum TG3. Other cultivated species showed a 16S rRNA similarity level of no more than 80%. These values indicate a class-level position of ACht6-1 in the candidate phylum TG3 (Yarza et al., 2014). Considering uncultured bacteria, ACh6-1 and related environmental clones formed a distinct branch within TG3, clustering with its subphylum S1 (**Figure 3A**).

To further characterize the phylogenetic affiliation of ACht6-1, the genome data of ACht6-1 and *Chv. alkaliphilus* ACht1 were used alongside with that from other organisms for phylogenetic analysis employing sequences of ribosomal proteins (**Figure 3B**). The obtained phylogeny is consistent with one resulted from 16S rRNA sequences and show that ACht6-1 and *Chv. alkaliphilus* ACht1 form a distinct deep branch together with *F. succinogenes* within a “superphylum” branch including the phyla *Fibrobacteres*, *Gemmatimonadetes*, *Chlorobi, Ignavibacteriae*, and *Bacteroidetes*.

To help further characterize phylogenetic positioning of ACht6-1, a taxonomy distribution analysis was carried out by screening all ACht6-1 protein-coding genes against the NCBI non-redundant database (Koonin and Wolf, 2008). For every gene producing a significant BLASTP hit, the taxonomy of its best homolog at the phylum level was recorded. The number of such best hits was counted for each phylum (**Table 1**). As expected, ACht6-1 proteins had most of their best matches in the *Fibrobacteres* (29.8%), particularly, in *Chv. alkaliphilus* (28.0%), followed by *Proteobacteria* (27.6%), *Firmicutes* (12.9%), and *Bacteroidetes* (6.9%). However, upon exclusion of *Chv. alkaliphilus* from this analysis, the ACht6-1 proteins appeared to have their best matches in different phylum-level bacterial lineages, *Proteobacteria* (35.4%), *Firmicutes* (19.1%), *Bacteroidetes* (9.5%), *Spirochaetes* (5.7%), and *Fibrobacteres* (5.2%). Even assuming the apparent bias due to unequal representation of different phyla in sequence databases, these results show that ACht6-1 and *Chv. alkaliphilus* are genetically distant from each other and even more distant from the other lineages in *Fibrobacteres*.

**Table 1:** The taxonomic distribution analysis of the ACht6-1 proteome.

Lineage	Best BLASTP hits (%)	
	
	with *Chv. alkaliphilus*	without *Chv. alkaliphilus*
Proteobacteria	27.6	35.4
Firmicutes	12.9	19.1
Bacteroidetes	6.9	9.5
Spirochaetes	4.3	5.7
Fibrobacteres	29.8	5.2
Cyanobacteria	2.8	3.2
Planctomycetes	1.7	2.5
Chlorobi	1.7	2.4
Acidobacteria	1.3	1.7
Verrucomicrobia	1.3	1.6
Chloroflexi	1.2	1.5
Actinobacteria	1.0	1.5
Nitrospirae	0.7	1.1
Deferribacteres	0.7	1.0
Archaea	2.2	3.0
Others	3.9	5.6


These data altogether support, on a whole-genome scale, that ACht6-1 and *Chv. alkaliphilus* ACht1 form two high-level taxa that should be classified as two new classes within the phylum *Fibrobacteres*.

### Chitin Degradation Pathways

**FIGURE 4:**
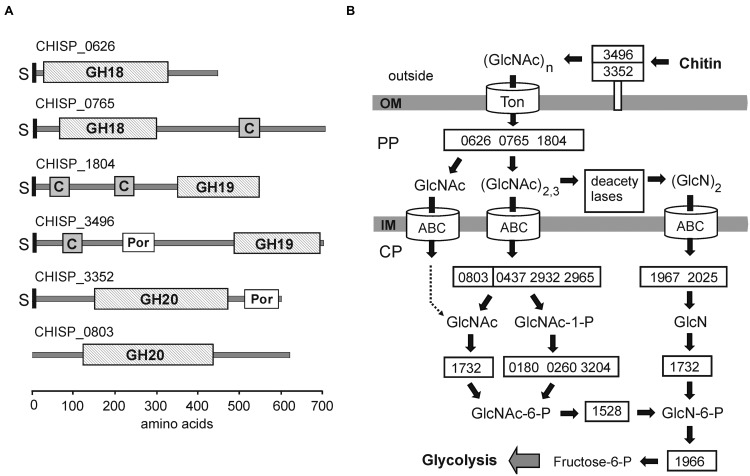
**Chitin degradation in ACht6-1.** **(A)** Schematic representation of domain structure of the GH18, GH19, and GH20 family hydrolases from ACht6-1. C, ChiC chitin-binding domain; Por, Por secretion system C-terminal sorting domain; GH, glycoside hydrolase catalytic domain; S, signal peptide. Domain localization is shown according to results of BLASTP searches against NCBI’s conserved domain database. The bar at the bottom of the figure showed coordinates of the amino acid residues in the predicted proteins. **(B)** Predicted pathways for degradation, transport and metabolism of chitin by strain ACht6-1. Enzymes are given gene identifiers from ACht6-1 when possible. Abbreviations: OM, outer membrane; PP, periplasm; CP, cytoplasm.

The phenotypic hallmark of ACht6-1 is its ability to utilize as a growth substrate insoluble chitin, but not soluble chito-oligomers or *N*-acetyl glucosamine, or any other polymers. The predicted chitin degradation pathway of ACht6-1 (summarized in **Figure 4**) is mostly similar to that found in *Chv. alkaliphilus* (Sorokin et al., 2014), in spite of limited sequence similarity of the enzymes involved.

The extracellular hydrolysis of chitin could be performed by a GH19 family endochitinase CHISP_3496. It is a 706-amino-acid (aa) protein carrying a N-terminal secretion signal predicted by SignalP 3.0, a ChiC chitin-binding domain, a Por sorting domain and a GH19 catalytic domain at the C-terminus (**Figure 4**). Since the presence of Por domains in secreted proteins indicate their linking to the outer membrane (Shoji et al., 2011; Slakeski et al., 2011), this chitinase is probably associated with cell surface. The BLASTP search against GenBank revealed the extracellular chitinase Calk0031 of *Chv. alkaliphilus* ACht1 as the best hit (39% identity along C-terminal 542-aa region), although this enzyme lacks a chitin-binding domain. Chitinases with a similar GH19 catalytic domain were identified among *Bacteroidetes* and *Firmicutes* (see **Figure S2** in the Supplemental Material). The search for other chitin degrading enzymes from GH families 18, 19, and 20, carrying both an N-terminal signal peptide and a Por sorting domain and thus probably associated with cell surface, revealed only GH20 family protein CHISP_3352. This enzyme, annotated as β-*N*-acetylglucosaminidase, could hydrolyse chitodextrins and produce *N*-acetyl-β-glucosamine, as suggested for *N*-acetyl-glucosaminidase Hex20A in *Saccharophagus degradans* (Hutcheson et al., 2011). The presence of this enzyme could explain high level of acetylglucosaminidase activity detected for ACht6-1 cells (see above). The GH20 family glycoside hydrolases are missing in *Chv. alkaliphilus* ACht1. It is possible that endochitinase CHISP_3496 and β-*N*-acetylglucosaminidase CHISP_3352 could act synergistically to degrade insoluble chitin.

Subsequent hydrolysis of oligosaccharides to shorter oligomers and GlcNAc probably proceeds in the periplasme. Two GH18 chitinases, CHISP_0626 and CHISP_0765, and the GH19 enzyme CHISP_1804, may account for the hydrolysis of chito-oligosaccharides into trimers and dimers of GlcNAc (Keyhani and Rosema, 1996). These three chitinases carry a typical type II-dependent secretion signal, but lack a Por sorting domain, indicating their export from the cytoplasm to the periplasm only. CHISP_0765 and CHISP_1804 enzymes also contain a ChiC chitin-binding domain (**Figure 4**). Biochemical characterization of recombinant CHISP_0765 and CHISP_1804 confirmed that these enzymes are endochitinases active with insoluble chitin and chitooligosaccharides longer than chitobiose (our unpublished data). CHISP_0765 hydrolyzes chitin and chitooligosaccharides to GlcNAc, chitobiose and chitotriose, while GlcNAc and chitobiose were detected for CHISP_1804. Both enzymes can bind to chitin consistent with the presence of ChiC domains.

Upon import into the cytoplasm, (GlcNAc)_2,3_ oligosaccharides can be digested by GH20 family *N*-acetylglucosaminidase (CHISP_0803) to produce chitobiose and GlcNAc. *N*,*N*′-diacetylchitobiose phosphorylase (CHISP_0437, CHISP_2932, CHISP_2965) can hydrolyse chitobiose and generate GlcNAc and GlcNAc-1-P (Park et al., 2000). Then, *N*-acetylglucosamine-1-P-mutase (CHISP_0180, CHISP_0260, CHISP_3204) makes GlcNAc-6-P from GlcNAc-1-P. ATP-dependent kinase (CHISP_1732) can phosphorylate GlcNAc also producing GlcNAc-6-P. At the final step, *N*-acetylglucosamine-6-phosphate deacetylase (CHISP_1528) and glucosamine-6-phosphate deaminase (CHISP_1966) transforms the GlcNAc-6-P into fructose-6-P that enters central carbohydrate metabolism.

The presence of polysaccharide deacetylases (15 candidate genes) indicate that an alternative pathway of utilization of chitin oligomers (Tanaka et al., 2003) may exist in ACht6-1 (**Figure 4**). Thirteen of them carry an N-terminal secretion signal, indicating their export from the cytoplasm. Periplasmic deacetylases can deacetylate GlcNAc dimers to (GlcN)_2_. Upon import into the cytoplasm, the β-1,4 linkage in the dimer could be hydrolysed by the GH9 family chitobiases CHISP_1967 and CHISP_2025. In the cytoplasme the ATP-dependent kinase CHISP_1732 can phosphorylate GlcN and then it can enter the Embden–Meyerhof pathway as described above. Interestingly, four putative chitin deacetylases carried both an N-terminal signal peptide and a Por sorting domain and could act at the cell surface, suggesting that chitin deacetylation could also proceed extracellularly.

The chitin utilization strategy of ACht6-1 appeared to be quite similar to that of *Chv. alkaliphilus* (Sorokin et al., 2014), although some functions are performed by non-orthologous enzymes (e.g., GH9 and GH2 family chitobiases in ACht6-1 and *Chv. alkaliphilus*, respectively). The most notable difference is that only intact cells of *Chv. alkaliphilus* could hydrolyze chitin, while lysis of ACht6-1 cells did not affect chitin hydrolysis. Three out of four chitinases present in ACht6-1, including the extracellular CHISP_3496, contain chitin-binding domains, while *Chv. alkaliphilus* chitinases lacks sugar-binding domains and thus this bacterium should rely only on a direct cell-mediated contact with chitin particles. In addition, *Chv. alkaliphilus* lacks the extracellular β-*N*-acetylglucosaminidase that could act synergistically with endochitinase in ACht6-1. Although ACht6-1 cells also attaches to insoluble chitin particles, the chitinolytic capabilities of this strain could be more versatile.

### Capacities for the Degradation of Other Polysaccharides

In spite of the growth-based observation that ACht6-1 is able to grow only on insoluble chitin, its genome contains more than 40 genes encoding carbohydrate-active enzymes (Cantarel et al., 2009) representing 16 families of glycoside hydrolases (see **Table S2** in the Supplemental Material). Besides glycoside hydrolases, numerous glycosyl transferases that could play a primary role in oligo- and polysaccharides formation are encoded. The number and diversity of glycoside hydrolases in ACht6-1 are much higher than in *Chv. alkaliphilus* (20 genes/12 families), but less than in specialized polysaccharide degrading bacteria such as *F. succinogenes* (134 genes/49 families; Suen et al., 2011) and *M. roseus* (100 genes/31 family; Kadnikov et al., 2013).

**FIGURE 5:**
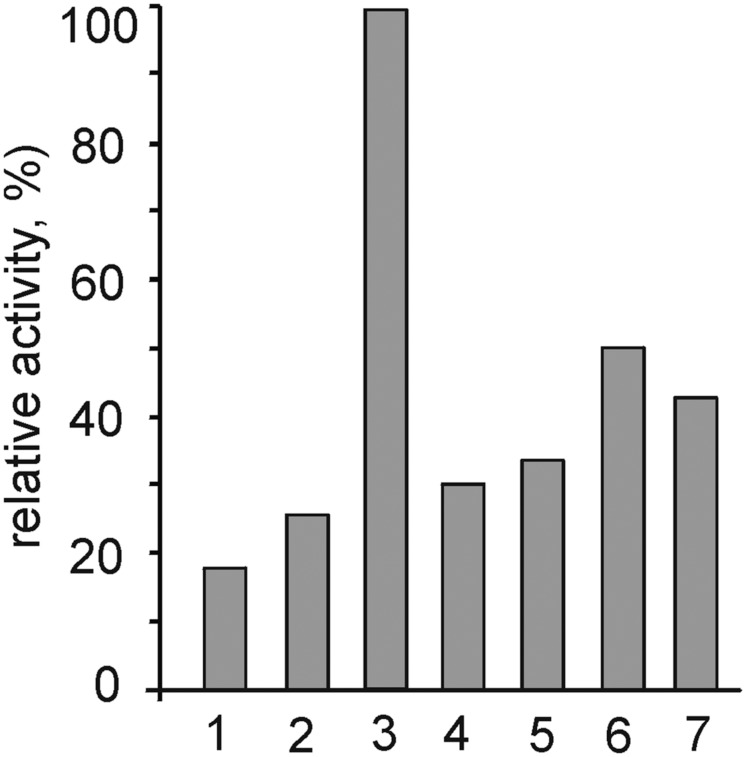
**Hydrolytic activities of ACht6-1 crude cell extract towards different polysaccharides.** The following substrates were tested: 1, chitin; 2, chitosan; 3, carboxymethyl cellulose; 4, microcrystalline cellulose; 5, starch; 6, beech-wood xylan; 7, birch-wood xylan.

The capacities of ACht6-1 to degrade different polysaccharides were evaluated by assaying the digestion of cellulose, amorphous chitosan, starch, and xylan by ACht6-1 cell extracts. Carboxymethyl cellulose (test substrate for the β-1,4-endoglucanase activity) was most efficiently hydrolyzed, and lower activities were observed towards starch, microcrystalline cellulose and chitosan (**Figure 5**). Despite the fact that ACht6-1 failed to grow with all these polymers, the cells grown with chitin showed positive results for amylase and β-1,4-endoglucanase in agar-diffusion assay (see **Figure S3** in the Supplemental Material). This assay also revealed proteolytic activity, although the organism was not able to grow on proteinaceous substrates.

Endo-1,4-β-glucanases of GH5 and GH9 families could perform the extracellular hydrolysis of cellulose. A number of these hydrolases in addition to N-terminal signal peptides comprise carbohydrate binding modules (CBMs) enabling their binding to substrates. The most likely candidates are the GH5 family endoglucanase CHISP_1332, containing a CBM4/CBM9 domain and a C-terminal Por domain, and the GH9 family enzyme CHISP_2683, containing the CBM6 and Por domains. Intracellular hydrolysis of cellobiose may be performed by β-glucosidase (CHISP_3415) and cellobiose phosphorylases (CHISP_0437, CHISP_2932, CHISP_2965).

Hydrolases of the GH8 family could cut the β-1,4 linkages in such polymers as chitosan, xylan, lichenan, and β-1,4 glucan. Five GH8 enzymes were identified in the ACht6-1 genome. Four hydrolases of this family are predicted to have signal peptides, and two of them contain a C-terminal Por sorting domain, suggesting their involvement in cell-linked hydrolysis of polysaccharides. Two GH16 family hydrolases, CHISP_1173 and CHISP_1878 could hydrolyze polysaccharides with β-1,3-1,4 linkages such as laminarin.

The results of this genome analysis are consistent with the observed inability of ACht6-1 to grow on alpha-linked polysaccharides like starch. Although at least six alpha-amylases are encoded, all of them lack a signal peptide and, probably, operate inside the cell in pathways related to the synthesis and degradation of storage polysaccharides (e.g., glycogen).

Overall, the actual role of carbohydrate-active hydrolases encoded in the ACht6-1 genome is unclear in view of the inability of ACht6-1 to grow on polysaccharides other than chitin. One of the possible function of these glycoside hydrolases is to “clean” chitin-containing biomass, making it accessible for hydrolysis similar to the strategy employed by *F. succinogenes* for access to cellulose (Ramson-Jones et al., 2012). Alternatively, some of these enzymes may play a primary role in the synthesis rather than hydrolysis of extracellular polysaccharides.

### Central Metabolic Pathways

ACht6-1 is an obligatory anaerobic fermentative bacterium producing H_2_, CO_2_, acetate, glycerol, ethanol, and formate as fermentation end products. Consistently, we found no pathways for autotrophic metabolism, as well as aerobic or anaerobic respiration in its genome, including proton-translocating NADH-dehydrogenase complexes and terminal reductases for respiration processes.

Similar to its fermentative relatives, *Chv. alkaliphilus* and *F. succinogens*, the ACht6-1 genome contain full set of genes of Embden–Meyerhof–Parnas (EMP) pathway. Contrary to *Chv. alkaliphilus*, however, the pentose phosphate pathway in ACht6-1 seems to be incomplete since the genes, coding for two key enzymes, transldolase, and 6-phosphogluconolactonase, were not identified. Pyruvate produced in the EMP pathway is decarboxylated by two pyruvate:feredoxin oxidoreductases (POR) to acetyl-CoA. The first POR is a fused enzyme (CHISP_0411) comprising α, β, γ, and δ subunits, also found in *Chv. alkaliphilus*; the second POR is a multi-subunit enzyme encoded by genes CHISP_1600-CHISP_1604. In addition ACht6-1 can produce acetyl-CoA and formate through the activity of pyruvate-formate lyase (CHISP_3535) and a pyruvate-formate lyase activating enzyme (CHISP_3536). Conversion of acetyl-CoA to acetate with the generation of ATP is probably performed in a one-step reaction by acetyl-CoA synthetase (CHISP_0439) rather than in a two-stage reaction catalyzed by phosphate acetyltransferase and acetate kinase, as in *Chv. alkaliphilus*, since the corresponding genes are missing in the ACht6-1 genome. Another fermentation product, ethanol, could be produced via aldehyde dehydrogenase (CHISP_1606) and several alcohol dehydrogenases (CHISP_2518, etc.).

The tricarboxylic acid (TCA) cycle in ACht6-1 is incomplete, lacking succinyl-CoA synthetase. Two TCA enzymes, succinate dehydrogenase/fumarate reductase and succinyl-CoA synthetase are also missing in *Chv. alkaliphilus*, while *F. succinogens* lacks 2-oxoglutarate ferredoxin oxidoreductase and succinyl-CoA-synthetase (Suen et al., 2011). *F. succinogenes* utilizes the reductive branch of its incomplete TCA for the generation of its main fermentation product, succinate. Oxaloacetate feeding this pathway can be produced from pyruvate and phosphoenolpyruvate by pyruvate carboxylase and phosphoenolpyruvate carboxykinase, respectively (Suen et al., 2011). These enzymes were not identified in the ACht6-1 genome, which could explain why succinate was not found among the main fermentation products of this bacterium.

The ACht6-1 genome contains two gene clusters that encode two different cytoplasmic [Fe-Fe] hydrogenases. The first cluster (CHISP_2841-2845) encodes a heterotetrameric enzyme similar to NADH-dependent hydrogenase from *Thermoanaerobacter tengcongensis* (Soboh et al., 2004). The subunits HydA (catalytic), HydB (NuoF-like), and HydC (NuoE-like) are also similar to corresponding subunits of the heterotrimeric bifurcating [Fe–Fe] hydrogenase from *Thermotoga maritima*, in which the exergonic oxidation of ferredoxin is used to drive the unfavorable oxidation of NADH to produce H_2_ (Schut and Adams, 2009). Therefore, HydABCD hydrogenase of ACht6-1 probably uses NADH and reduces ferredoxin for hydrogen turnover. The second hydrogenase cluster in ACht6-1 (CHISP_3024-3028) is similar to an operon encoding the ferredoxin-dependent [Fe–Fe] hydrogenase Hfs in *Thermoanaerobacterium saccharolyticum* (Shaw et al., 2009). The activity of two [Fe–Fe] hydrogenases collectively able to use both NADH and reduced ferredoxin generated during anaerobic fermentation will link their oxidation and the reduction of protons to H_2_. Consistently, hydrogen was found among the fermentation products.

### Generation of a Transmembrane Ion Gradient

Being a haloalkaliphile, ACht6-1 is required to maintain physiological levels of Na^+^ inside the cell by extrusion of Na^+^ ions across the cytoplasmic membrane. Analysis of the ACht6-1 genome revealed an operon *rnfCDGEAB* (CHISP_2598-2603), that was predicted to encode the membrane-bound electron transport complex Rnf. Similar complex was identified also in *Chv. alkaliphilus.* It was proposed (Biegel and Müller, 2010; Biegel et al., 2011) that the Rnf complex in acetogens could act as ferredoxin:NAD^+^ oxidoreductase generating transmembrane ion gradient. Rnf oxidize ferredoxin simultaneously reducing NAD^+^ and performing the transport of Na^+^ or H^+^ ions across the cytoplasmic membrane. Like in *Chv. alkaliphilus*, the sodium transmembrane ion gradient in ACht6-1 may be generated by a predicted oxaloacetate decarboxylase (CHISP_0486-0488), membrane-linked enzyme functioning as a primary sodium pump in some obligate anaerobes (Buckel, 2001).

The physiological levels of Na^+^ inside the cell may be also maintained due to activity of V-type ATPase encoded by genes CHISP_1230-CHISP_1236 (subunits *KIDBACE*). As expected for an anaerobic haloalkaliphile, this enzyme complex appears to use the ATP hydrolysis for the transportation of Na^+^ instead of H^+^, as suggested by the Na^+^-binding motif (Murata et al., 2005; Kawano-Kawada et al., 2012) in the K subunit.

An additional enzyme, not found in *Chv. alkaliphilus*, could also contribute to maintaining the transmembrane ion gradient. This is the predicted integral membrane V-type pyrophosphatase (CHISP_2394). These enzymes could couple the hydrolysis of biosynthetic waste pyrophosphate to the translocation of protons or sodium ions. The absence of lysine in the GNXX(K/A) signature sequence suggests that CHISP_2394 belongs to a family of prokaryotic Na^+^ translocating pyrophosphatases (Malinen et al., 2007).

### Proposal for a New Class *Chitinispirillia* Within the Phylum *Fibrobacteres*

Strain ACht6-1 represents the second natronophilic chitinotrophic member of the candidate phylum TG3 found in hypersaline alkaline lakes. Despite a significant phylogenetic distance to *Chv. alkaliphilus*, both groups share a similar exotic lifestyle by exclusive utilization of insoluble chitin for fermentative growth under haloalkaline conditions employing cell-bound chitinases for chitin degradation with the formation of identical fermentation products. However, a number of important phenotypic differences discriminate these two groups of chitinotrophic haloalkaliphiles, including a much lower salt tolerance and different cell morphology of ACht6-1 and a major difference in the polar lipid and PLFA profiles.

According to the phylogenetic analysis of both 16S rRNA sequences and concatenated ribosomal protein, ACht6-1 formed a novel class-level lineage in the candidate phylum TG3, representing its subphylum S1, as defined by Hongoh et al. (2006). Originally, TG3 was proposed as a distinct phylum comprising environmental 16S RNA sequences initially identified in the termites gut and subsequently also in different aquatic environments (Hongoh et al., 2006). Subsequently, TG3 was suggested to be reclassified as a distinct lineage within *Fibrobacteres* (He et al., 2013). Later, the haloalkaliphic species *Chv. alkaliphilus*, the first cultured representative of TG3, was assigned to a new class *Chitinivibrionia* within the phylum *Fibrobacteres*, corresponding to subphylum S2 of TG3 (Sorokin et al., 2014). The phylogenetic analysis of ACht6-1 supports these classifications.

Genomic analysis showed that strain ACht6-1, like *Chv. alkaliphilus*, specializes in the fermentative metabolism of chitin and, possibly, the degradation of some other polysaccharides. The reconstructed central metabolism of ACht6-1 and *Chv. alkaliphilus* revealed the EM pathways enabling the fermentation of sugars. The generated reduced products, NADH and reduced ferredoxin, could be re-oxidized by two [Fe–Fe]-family hydrogenases. Metabolic pathways resulted in the production of ethanol and glycerol could also contribute to the oxidation of NADH. Both bacteria lack aerobic or anaerobic respiration pathways and have a limited ability to generate transmembrane ion gradients employing an Rnf-like complex and oxaloacetate decarboxylase. Adaptation to a haloalkaliphilic environment is evidenced by the possible use of sodium ions instead of protons in bioenergetics, as indicated by the presence of a Na^+^ translocating V-type ATPase and, in the case of ACht6-1, a Na^+^ translocating pyrophosphatase. Osmolyte biosynthesis pathways are present in both bacteria, although different solutes are produced, i.e., glycine betaine by ACht6-1 and ectoine by *Chv. alkaliphilus.* Interestingly, in spite of similar metabolic capabilities, the ACht6-1 genome is much bigger than that of *Chv. alkaliphilus* (4.4 Mbp vs. 2.6 Mbp). There are several contributors to this difference. There are unique traits encoded by the ACht6-1 genome like the Rhs family toxins and the type VI secretion system, specific integrated phages and mobile elements, etc. The second reason is the expansion of several gene families, like carbohydrate-active enzymes and ABC-transporters, in the ACht6-1 genome. Overall, paralogous genes (>25% amino acid sequence identity along >70% of the gene length) account for about 0.91 and 0.37 Mbp in the genomes of ACht6-1 and *C. alkaliphilus*, respectively.

The genome and phenotypic properties of *F. succinogenes*, the only other sequenced representative of the phylum *Fibrobacteres* besides ACht strains, supports the assignment of these organisms to a single phylum. Like ACht6-1 and *Chv. alkaliphilus*, *F. succinogenes* inhabits an organic-rich environment (animal rumen) and relies on anaerobic fermentation of polysaccharides (Ransom-Jones et al., 2012; Spain et al., 2012). They employ peculiar mechanisms of polysaccharide utilization based on direct contact of the cells with insoluble polymers, i.e., crystalline cellulose (*F. succinogenes*) or chitin (ACht6-1 and *Chv. alkaliphilus*). Moreover, all three bacteria can hydrolyze various polysaccharides but can utilize the products of hydrolysis of only a single specific substrate, either cellulose (*Fibrobacter*) or chitin (*Chitinivibrio* and *Chitinispirillum*). Sugars fermentation proceeds via the EMP pathway, while the TCA cycle is incomplete and there are no pathways for aerobic or anaerobic respiration, including proton-translocating NADH-dehydrogenase complexes and terminal membrane-bound reductases. An overview of the phenotypic and genomic features of ACht6-1, *Chv. alkaliphilus*, and *F. succinogenes* is shown in **Table 2**.

**Table 2:** Comparative phenotypic and genomic features of ACht6-1, *Chv. alkaliphilus*, and *F. succinogenes.*

Property	Strain ACht6-1	*Chitinivibrio alkaliphilus* ACht1	*Fibrobacter succinogenes* S85
Isolation source	Soda lake	Soda lake	Cow rumen
pH range for growth (optimum)	8.5–10.5 (9.5–10)	8.5–10.6 (9.5–10)	Neutrophilic
Salinity range for growth (optimum)	0.4–1.75 (0.6–1.0)	0.6–3.5 (1.0–1.5)	Freshwater
Optimum growth temperature	33–35°C	35–37°C	37°C
Relation to oxygen	Obligate anaerobe	Obligate anaerobe	Obligate anaerobe
Cell morphology	Spirillum, polar flagellum, lipid cysts	Vibrio, polar flagellum, intracellular bodies	Nonmotile rods
Dominant fatty acids in polar membrane lipids	ai15:0, C14:0, C16:0	16:0, C16:1ω7	13:0, 13:1, 15:0
Dominant polar lipids	Phosphoglycerol, orhnithine lipid	Phosphatidylglycerol	Phosphatidylethanolamine
Genome size (Mb)	4.4	2.6	3.8
GC (%)	42.9	46.2	48.1
Number of genes	3827	2346	3188
Key physiology	Sugar fermentation	Sugar fermentation	Sugar fermentation
Growth substrates	Chitin	Chitin	Cellulose, cellobiose, glucose, lactose (weak)
Hydrolytic activity (substrates)	Chitin, xylan, starch, carboxymethyl cellulose	Chitin, xylan, carboxymethyl cellulose	Cellulose, xylan, glucomannan, xyloglucan, pectin, inulin, phlein
GH families in the genomes	GH 1, 5, 8, 9, 13, 15, 16, 18, 19, 20, 31, 43, 57, 77, 81, 94	GH 1, 2, 5, 8, 9, 10, 13, 16, 18, 19, 57, 77, 84, 94	GH 2, 3, 5, 8, 9, 10, 11, 13, 16, 18, 23, 26, 27, 30, 43, 44, 45, 51, 53, 54, 57, 74, 77, 94, 95, 105, 116, 127
Fermentation products	H_2_, formate, acetate, ethanol, glycerol	H_2_, formate, acetate, ethanol, lactate, glycerol	Acetate, succinate, formate
Glycolytic pathway (s)	EMP	EMP, PP	EMP, PP
Tricarboxylic acid (TCA) cycle	Incomplete, lacking succinyl-CoA synthetase	Incomplete, lacking succinate dehydrogenase and succinyl-CoA synthetase	Incomplete, lacking α-ketoglutarate dehydrogenase and succinyl-CoA-synthetase
Hydrogenases	Two [Fe–Fe] hydroganases (cytoplasmic)	Two [Fe–Fe] hydroganases (cytoplasmic)	–
Electron transport chain	–	–	–
Rnf complex	+	+	–


*EMP, Embden–Meyerhof–Parnas; PP, pentose phosphate pathway.*

Based on the results of phenotypic and phylogenetic analysis, ACht6-1 is suggested to be assigned to a novel genus and species on the basis of which we also propose to form a new family *Chitinispirillaceae*, a new order *Chitinispirillales* and a new class *Chitinispirillia*. Despite the fact, that, nominally, the classes *Chitinivibrionia* and *Chitinispirillia* represent two subphyla of the candidate phylum TG3, formally, the appearance of culturable representatives in this large cluster of uncultured organisms changes its taxonomic status. We believe that it would be reasonable to include the candidate phylum TG3 into the phylum *Fibribacteres* based on the monophyly of these lineages and common lifestyle properties. This proposal is consistent with the recently published results of a phylogenomic study of the phylum *Fibrobacteres* (Abdul Rahman et al., 2016). Two novel deep lineages, based on cultured natronophilic chitinotrophs from hypersaline alkaline lakes, will then represent two former subphyla of the TG3 in the phylum *Fibribacteres* as two new classes.

#### Description of *Chitinispirillia* classis nov.

*Chitinispirillia* (Chi.ti.ni.spi.ril’lia N.L. n. *Chitinispirillum* type genus of the type order of the class; N.L. pl. n. *Chitinispirillia* a class of the genus *Chitinivibrio*). The class *Chitinispirillia* is defined on a phylogenetic basis by comparative 16S rRNA and complete genome sequence analysis of the type strain ACht6-1 isolated from hypersaline alkaline lakes and multiple uncultured representatives from terrestrial and marine habitats. Gram-negative bacteria. Type order: *Chitinispirillales* ord. nov.

#### Description of *Chitinispirillales* order. nov.

The description is the same as for the genus *Chitinispirillum*. Type family: *Chitinispirillaceae* fam. nov.

#### Description of *Chitinispirillaceae* fam. nov.

The description is the same as for the genus *Chitinispirillum*. Type genus: *Chitinispirillum* gen. nov.

#### Description of *Chitinispirillum* gen. nov.

*Chitinispirillum* (Chi.ti.ni.spi.ril’ lum N.L. n. *chitinum*, chitin; Gr. n. *spira* spiral; M. L. dim. neut. n. *Spirillum* a small spiral. M. L. neut. n. *Chitinispirillum* a spirillum utilizing chitin as substrate). Cells are motile, vibrio-shaped. Gram-negative. In old cultures forms lipid cysts. Fermentative anaerobe growing on chitin as the carbon and energy source. Moderately salt-tolerant obligate alkaliphile. Inhabits hypersaline alkaline lakes. The type species is *Chitinispirillum alkaliphilum*. Forms a deep phylogenetic lineage within the candidate division TG3 associated with the phylum *Fibrobacteres*.

#### Description of *Chitinispirillum alkaliphilum* sp. nov.

*alkaliphilum* (al.ka.li’phi.lum N.L. n. *alkali*, soda ash; N.L. adj. philum (from Gr. adj. philos -ê -on), friend, loving; N.L. adj. alkaliphilum, alkali-loving).

Cells are Gram-negative, loose spirilla, 0.5–0.8 μm × 2–10 μm, motile by a single lateral flagellum. The cell wall is unstable, an extensive cell lysis was observed in the stationary phase. Strictly anaerobic with fermentative metabolism. Colony formation was not observed. Utilizes chitin as the only growth substrate and produces H_2_, acetate, glycerol, ethanol, and formate as fermentation products. Mesophilic, with an optimum growth temperature at 33–35°C and maximum at 43°C. Obligately alkaliphilic with a pH range for growth from 8.5 to 10.5 (optimum at pH 9.5–10) and moderately salt-tolerant with a total Na^+^ range for growth from 0.4 to 1.75 M (optimum at 0.5–1 M). The major membrane polar lipids are phosphoglycerol and orhnithine lipid. The dominant membrane PLFA is ai15:0. The G + C content of the genomic DNA in the type strain is 43 mol%. The type strain, ACht6-1^T^ (=DSM 24539^T^ = UNIQEM U893^T^) was isolated from sediments of hypersaline alkaline lakes in Wadi el Natrun, Egypt. The accession number of 16S rRNA gene sequence of the type strain in GenBank is JF304644.

## Author Contributions

DS and NR designed the research project and wrote the paper; DS, AR, VG, JSD, and AM performed the research; DS, AR, VG, AB, AM, and NR analysed the data.

## Conflict of Interest Statement

The authors declare that the research was conducted in the absence of any commercial or financial relationships that could be construed as a potential conflict of interest.
