# Supplementary material for: Phenotypic and Genomic Properties of Chitinispirillum alkaliphilum gen. nov., sp. nov., A Haloalkaliphilic Anaerobic Chitinolytic Bacterium Representing a Novel Class in the Phylum Fibrobacteres
Source: Front Microbiol. 2016 Mar 31;7:407. doi: 10.3389/fmicb.2016.00407 (PMC4814513; doi:10.3389/fmicb.2016.00407)
Supplement: Supplementary file 1 [file Table_1.DOC]

**Table S1a**. Composition of PLFA in strain Acht6-1 grown at 30^o^C, pH 10 and 0.6 M total Na^+^ until late exponential phase.

| Fatty acids | Relative abundance (%) |
| --- | --- |
| C14:0 | 11.5 |
| iC14:0 | 1.0 |
| iC15:0 | 0.7 |
| aiC15:0 | 62.5 |
| C16:0 | 14.3 |
| iC16:0 | 6.7 |
| iC17:0 | 3.3 |

**Table S1b**. Composition and relative abundances^a^ and acyl/alkyl composition^b^ of membrane polar lipids in strain Acht6-1

| IPL**^c^** | Relative abundance | Acyl/alkyl moieties |
| --- | --- | --- |
| PG-dialkyl | ++ | 30:0, 31:0 |
| PG-alkyl/acyl | + | 30:0, 31:0 |
| PG-diacyl | ++ | 31:0, 33:0, 30:0, 33:0 |
| OL | +++ | 31:0, 32:0, 30:0, 33:0 |
| Unknown^d^ | + | n.d. |
| Unknown^e^ | + | n.d. |
| Phex | + | 31:0, 32:0 |

^a^ Abundance relative to major peak in the LC-MS chromatogram (+++, base peak; ++, 50-100% of base peak; +, 10-50% of base peak). Note that the mass spectral response factors for different IPL groups can be quite different.

^b^ The predominant composition is reported as the total number of carbon atoms of the alkyl and acyl moieties and the number of double bond equivalents. Mass spectral characterization does not allow to identify the branching of the acyl/alkyl moieties.

^c^ Listed in order of elution; PG, phosphoglycerol; OL, ornithine lipid; Phex, phosphohexose.

^d^ Unknown IPL characterized by *m/z* 1016

^e^ Unknown IPL characterized by *m/z* 755 and 769.
