# Supplementary material for: Phenotypic and Genomic Properties of Chitinispirillum alkaliphilum gen. nov., sp. nov., A Haloalkaliphilic Anaerobic Chitinolytic Bacterium Representing a Novel Class in the Phylum Fibrobacteres
Source: Front Microbiol. 2016 Mar 31;7:407. doi: 10.3389/fmicb.2016.00407 (PMC4814513; doi:10.3389/fmicb.2016.00407)
Supplement: Supplementary file 2 [file Table_2.DOC]

**Table S2.** Glycoside hydrolases predicted from the genome of strain ACht6-1.

| GH family | Gene | Signal peptide | Modular structure | In silico prediction |
| --- | --- | --- | --- | --- |
| 1 | CHISP_3415 | - | GH1 | beta-glucosidase |
| 5 | CHISP_0002 | - | GH5 | cellulase/endoglucanase |
|  | CHISP_1102 | + | GH5 – CBM4/9 | cellulase/endoglucanase |
|  | CHISP_1194 | + | CBM11-GH5 | cellulase/endoglucanase |
|  | CHISP_1332 | + | GH5 – CBM4/9-Por | cellulase/endoglucanase |
|  | CHISP_2154 | + | GH5-CBM4/9 | cellulase/endoglucanase |
|  | CHISP_2806 | - | GH5-CBM4/9-CBM4/9 | cellulase/endoglucanase |
|  | CHISP_2935 | - | GH5 | cellulase/endoglucanase |
| 8 | CHISP_0185 | + | GH8-Por | chitosanase/beta-glucanase |
|  | CHISP_1093 | + | GH8 | chitosanase/endoglucanase |
|  | CHISP_1832 | + | GH8-Por | chitosanase/endoglucanase |
|  | CHISP_3119 | + | GH8 | chitosanase/beta-glucanase |
|  | CHISP_3252 | - | GH8 | chitosanase/endoglucanase |
| 9 | CHISP_0645 | + | GH9-Por | endoglucanase |
|  | CHISP_1967 | - | E_set-GH9 | endoglucanase/ chitobiase |
|  | CHISP_2025 | + | E_set-GH9- F5/8 type C-Por | endoglucanase/ chitobiase |
|  | CHISP_2624 | + | E_set – GH9 | endoglucanase |
|  | CHISP_2683 | + | GH9-CBM6-Por | endoglucanase |
|  | CHISP_3067 | + | E_set-GH9 | endoglucanase |
| 13 | CHISP_2108 | - | GH13 (pfam00128) | alpha-amylase |
| 15 | CHISP_3018 | - | GH15 | glucoamylase |
| 16 | CHISP_1173 | + | GH16 | beta-glucanase |
|  | CHISP_1878 | + | GH16-CBM4/9-Por | beta-glucanase/ licheninase/ laminarinase |
| 18 | CHISP_0626 | + | GH18 | chitinase |
|  | CHISP_0765 | + | GH18-ChiC_BD | chitinase |
| 19 | CHISP_1804 | + | ChiC_BD-ChiC_BD-GH19 | chitinase |
|  | CHISP_3496 | + | ChiC_BD-Por_GH19 | chitinase |
| 20 | CHISP_0803 | - | GH20 | beta-N-acetylhexosaminidase |
|  | CHISP_3352 | + | GH20- Por | beta-N-acetylhexosaminidase |
| 31 | CHISP_2284 | - | GH31 | alpha-glucosidase |
| 43 | CHISP_0989 | - | GH43 | alpha-L-arabinofuranosidase/ beta-D-xylosidase |
| 57 | CHISP_0345 | - | GH57-DUF3536 | alpha-amylase |
|  | CHISP_0730 | - | GH57 | GH57 glycoside hydrolase |
|  | CHISP_1412 | - | GH57-DUF1957 | 1,4-alpha-glucan-branching enzyme |
|  | CHISP_1807 | - | GH57 | alpha-amylase |
|  | CHISP_2786 | - | GH57-DUF1957 | 1,4-alpha-glucan-branching enzyme |
|  | CHISP_2977 | - | GH57-DUF1926 | alpha-amylase |
|  | CHISP_2999 | - | GH57 | alpha-amylase |
| 77 | CHISP_1614 | - | GH77 | 4-alpha-glucanotransferase |
|  | CHISP_2011 | - | GH77 | 4-alpha-glucanotransferase |
|  | CHISP_2738 | - | GH77 | 4-alpha-glucanotransferase |
| 81 | CHISP_1879 | + | GH81-Por | endo-β-1,3-glucanase |
| 94 | CHISP_0437 | - | GH94 | cellobiose/chitobiose phosphorylase |
|  | CHISP_2932 | - | GH94-GDE_C | cellobiose/chitobiose phosphorylase |
|  | CHISP_2965 | - | GH94 | cellobiose/chitobiose phosphorylase |

Abbreviations: CBM, carbohydrate binding module; Por, Por secretion system C-terminal sorting domain; E_set, N terminal Early set domain associated with the catalytic domain of cellulase; ChiC_BD, chitin-binding domain (cd12215); F5/8 type C, discoidin domain (pfam00754); GDE_C, GDE_C domain (pfam06202); DUF, domain of unknown function.
