# Supplementary material for: Phenotypic and Genomic Properties of Chitinispirillum alkaliphilum gen. nov., sp. nov., A Haloalkaliphilic Anaerobic Chitinolytic Bacterium Representing a Novel Class in the Phylum Fibrobacteres
Source: Front Microbiol. 2016 Mar 31;7:407. doi: 10.3389/fmicb.2016.00407 (PMC4814513; doi:10.3389/fmicb.2016.00407)
Supplement: Supplementary file 3 [file Image_1.PDF]

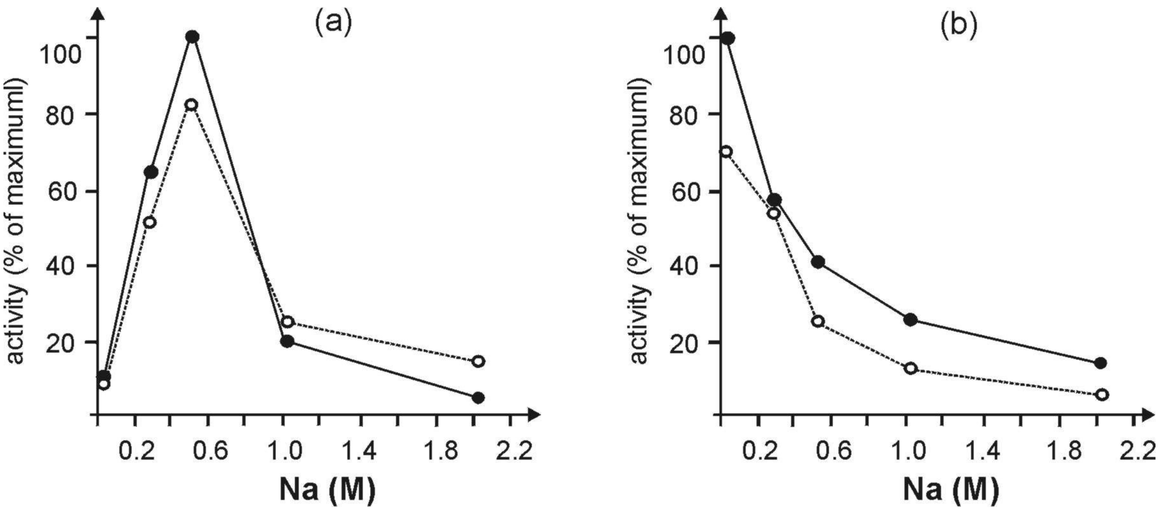

Figure S1. Influence of salt concentration on chitinolytic activities of strain ACht6-1.

Activities of whole cells (open circles) and cells lysed by sonication (closed circles) were measured with endochitinase substrate 4-MU-β-D-N, N′,N″-triacetylchitotriose (a) or with β-N-acetylglucosaminidase substrate 4-MU-N-acetyl-β-D-glucosaminide (b). Assays were performed at 30°C in 25 mM sodium phosphate buffer (pH 8.0) supplemented with NaCl (from 0 to 2 M). Activities are shown in relative units proportional to the amounts of liberated 4-MU.

The maximal activities (100%) were 0.04 U per mg of total cell protein with 4-MU-β-D-N, N′,N″-triacetylchitotriose and 5.8 U per mg of total cell protein with 4-MU-N-acetyl-β-D-glucosaminide. One unit (U) of activity was defined as the amount of enzyme required to liberate 1 μmol of 4-MU per min.
