# Supplementary material for: Phenotypic and Genomic Properties of Chitinispirillum alkaliphilum gen. nov., sp. nov., A Haloalkaliphilic Anaerobic Chitinolytic Bacterium Representing a Novel Class in the Phylum Fibrobacteres
Source: Front Microbiol. 2016 Mar 31;7:407. doi: 10.3389/fmicb.2016.00407 (PMC4814513; doi:10.3389/fmicb.2016.00407)
Supplement: Supplementary file 4 [file Image_2.PDF]

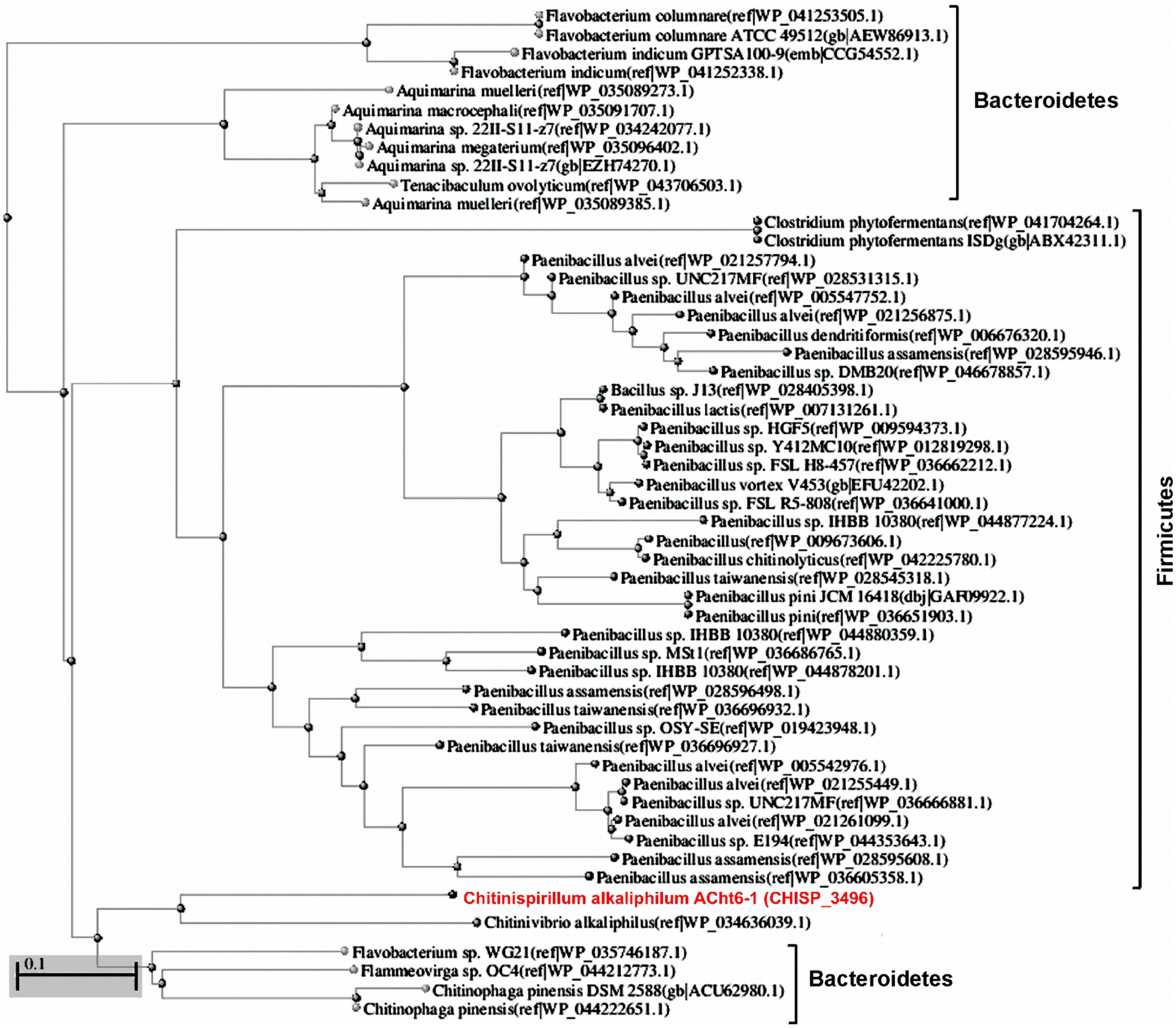

Figure S2. Phylogenetic relationships of the catalytic GH19 domain of extracellular chitinase CHISP_3496.

Neighbour joining tree based on the amino acid sequences of GH19 catalytic domains of CHISP_3496 (a.a. 495-700) and related enzymes. The tree was computed by NCBI BLASTP phylogenetic tree service using 5,000 best BLASTP hits. Only a fraction of the whole tree is shown. The scale bar represents the number of substitutions per site.
