# Supplementary material for: Phenotypic and Genomic Properties of Chitinispirillum alkaliphilum gen. nov., sp. nov., A Haloalkaliphilic Anaerobic Chitinolytic Bacterium Representing a Novel Class in the Phylum Fibrobacteres
Source: Front Microbiol. 2016 Mar 31;7:407. doi: 10.3389/fmicb.2016.00407 (PMC4814513; doi:10.3389/fmicb.2016.00407)
Supplement: Supplementary file 5 [file Image_3.PDF]

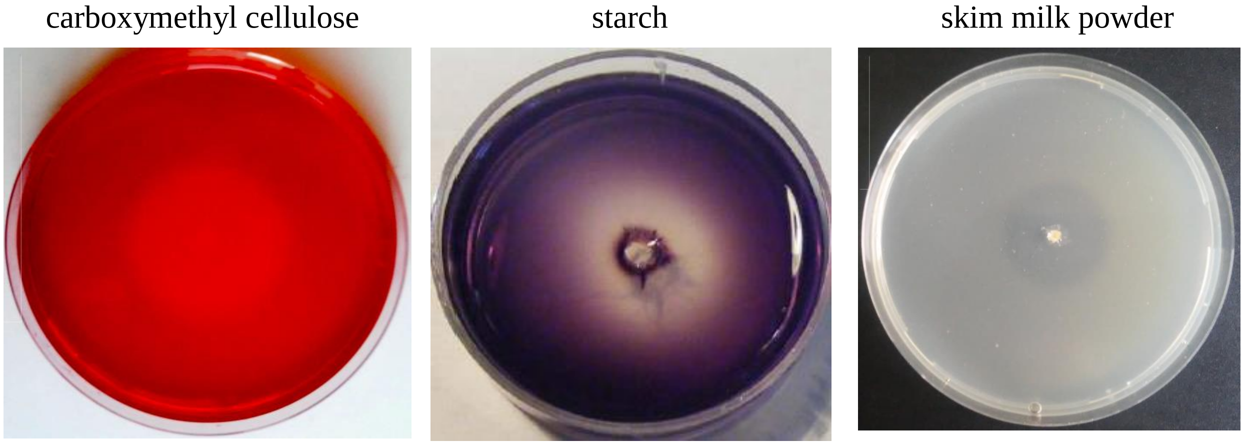

Figure S3. Agar-diffusion test for hydrolytic activity of strain ACht6-1 grown on chitin.

Cells grown on chitin were resuspended in soda buffer with pH 10 and 0.6 M Na^+ and stored frozen. Before the test the cells were disrupted by repeated freeze-sawing. Carboxymethyl cellulose, soluble starch, skim milk powder, lichenan, birch-wood xylan and beech-wood xylan, were used as substrates in 1% agarose plates prepared with mineral soda buffer at pH 10 and 0.4 M total Na^+. 500 μg (carboxymethyl cellulose and skim milk powder) or 250 μg (other substrates) of cell protein was loaded, and the plates were incubated at 30°C for 4 days. No activity was observed with two types of xylan and lichenan.
